# In-silico discovery of cancer-specific peptide-HLA complexes for targeted therapy

**DOI:** 10.1186/s12859-016-1150-2

**Published:** 2016-07-20

**Authors:** Ankur Dhanik, Jessica R. Kirshner, Douglas MacDonald, Gavin Thurston, Hsin C. Lin, Andrew J. Murphy, Wen Zhang

**Affiliations:** Regeneron Pharmaceuticals Inc, Old Saw Mill River Road, Tarrytown, NY USA

**Keywords:** Immunoinformatics, Bioinformatics, TCGA, GTEx, Cancer, Targeted therapy, Peptide-HLA

## Abstract

**Background:**

Major Histocompatibility Complex (MHC) or Human Leukocyte Antigen (HLA) Class I molecules bind to peptide fragments of proteins degraded inside the cell and display them on the cell surface. We are interested in peptide-HLA complexes involving peptides that are derived from proteins specifically expressed in cancer cells. Such complexes have been shown to provide an effective means of precisely targeting cancer cells by engineered T-cells and antibodies, which would be an improvement over current chemotherapeutic agents that indiscriminately kill proliferating cells. An important concern with the targeting of peptide-HLA complexes is off-target toxicity that could occur due to the presence of complexes similar to the target complex in cells from essential, normal tissues.

**Results:**

We developed a novel computational strategy for identifying potential peptide-HLA cancer targets and evaluating the likelihood of off-target toxicity associated with these targets. Our strategy combines sequence-based and structure-based approaches in a unique way to predict potential off-targets. The focus of our work is on the complexes involving the most frequent HLA class I allele HLA-A*02:01. Using our strategy, we predicted the off-target toxicity observed in past clinical trials. We employed it to perform a first-ever comprehensive exploration of the human peptidome to identify cancer-specific targets utilizing gene expression data from TCGA (The Cancer Genome Atlas) and GTEx (Gene Tissue Expression), and structural data from PDB (Protein Data Bank). We have thus identified a list of 627 peptide-HLA complexes across various TCGA cancer types.

**Conclusion:**

Peptide-HLA complexes identified using our novel strategy could enable discovery of cancer-specific targets for engineered T-cells or antibody based therapy with minimal off-target toxicity.

**Electronic supplementary material:**

The online version of this article (doi:10.1186/s12859-016-1150-2) contains supplementary material, which is available to authorized users.

## Background

Peptide-HLA (Human Leukocyte Antigen) class I complexes displayed on the cell surface play an important role in the T-cell mediated immune response [1, 2]. The approximately 9-residue long peptides originate from proteins that are digested by the proteasome inside the cell [3, 4]. Depending on whether the T-cell receptor recognizes a peptide as self or non-self, an immune response may be initiated [5]. Peptide-HLA complexes displayed specifically on the surface of cancer cells provide an excellent opportunity to develop targeted cancer therapeutics [6]. Such therapeutics could include engineered T-cells or “TCR-like” antibodies [7]. To inhibit the unwanted proliferation of cancer cells, the engineered T-cells are designed to express a receptor that can bind to peptide-HLA complexes on the surface of the cancer cells, thereby effecting cytotoxicity [8–11]. “TCR-like” antibodies may also be designed to link peptide-HLA complexes on cancer cells to cytotoxic T-cells [12–15]. Although these therapeutic technologies are under constant development, several studies have shown that they are very effective in killing cells [8, 16–18]. Extreme caution is, therefore, required to develop these therapeutic molecules so that they specifically kill only cancer cells and not cells from essential, normal tissues (a tissue that cannot be sacrificed in the course of cancer therapy is considered essential).

For precisely killing the cancer cells using the engineered T-cells or antibodies, it is important to target peptide-HLA complexes that are present “only” on the surface of cancer cells [19, 20]. The presence of the target complexes on the surface of cells in essential, normal tissues could easily result in adverse events [17, 21–23]. If the target peptides, i.e., peptides in the target complexes, are derived from genes that have low or no expression in the essential, normal tissues, then the obvious implication is that the adverse events due to toxic cross-reactivity can be avoided. However, recent research has demonstrated that avoiding the toxic cross-reactivity involving peptide-HLA targets is not that straightforward, and therefore, extremely challenging [24, 25].

Several clinical trials involving engineered T-cells targeted against peptide-HLA complexes recently have reported fatal adverse events. In one clinical trial [17], 9 cancer patients received engineered T-cells targeting a peptide-HLA complex involving peptide KVAELVHFL from MAGEA3 and HLA-A*02:01. Melanoma antigen A3 or MAGEA3 is a cancer testis antigen, expressed in tumor tissue and not significantly expressed in essential, normal tissues. Therefore, targeting of peptides from MAGEA3 and many other genes in the MAGE family was considered unlikely to result in off-target toxicity. However, beginning 1-2 days post infusion, 3 patients experienced mental status changes, and 2 out of 3 patients lapsed into coma and subsequently died. It was later discovered that the fatal toxicity most likely occurred due to off-target effects involving MAGEA12 (a melanoma antigen gene expressed in brain) peptide KMAELVHFL, which also binds to HLA-A*02:01. In another clinical trial [23], two melanoma patients, who received engineered T-cells designed to bind to a MAGEA3 peptide (amino acid sequence EVDPIGHLY) in complex with HLA-A01:01 suffered fatal cardiac toxicity. Subsequent to the fatal adverse events, it was discovered that a peptide-HLA complex (ESDPIVAQY-HLA-A*01:01) similar to the target complex was the most likely culprit of off-target toxicity. The peptide is contained in a gene called Titin (TTN) that is highly expressed in heart and skeletal muscle.

The outcome from these two clinical trials suggests that extreme caution is required when targeting peptide-HLA complexes. As suggested by the investigations performed after the clinical trials described above, a peptide that has between 5 and 8 identical amino acids can potentially cause off-target effects. Therefore, there are two critical factors when considering targeting of HLA-peptide complexes. First, expression of the peptide in the target complex should be cancer-specific. Second, peptides that may form a cross-reacting complex (i.e., containing a peptide similar to that of the target) should not be present in essential, normal tissues. Attempts to predict cross-reacting “off-targets” generally include a BLAST search of the human peptidome to find sequences similar to the target peptide, followed by selection of the most similar peptides predicted to bind the HLA allele of interest that are ubiquitously expressed [22, 23]. The recently published Expitope webserver is designed to perform these steps in a systematic fashion [26]. The webserver combines BLAST searches (to identify similar peptides), and various prediction tools that predict whether the similar peptides will form complexes with the HLA allele of interest. The webserver also returns expression levels of the peptide in cancer cell lines and normal tissues from Illumina Human Body Map (ArrayExpress accession: E-MTAB-513; ENA archive: ERP000546).

In contrast to the scope of the Expitope webserver, our work focuses on the identification of cancer-specific peptide-HLA targets using gene expression data from cancer tissues and essential, normal tissues. Here we describe a novel computational strategy that analyzes the canonical human peptidome, identifies potential peptide-HLA cancer targets, and then prioritizes them based on the likelihood of cross-reactivity that could be associated with them. First, all possible 9-mer peptides in the canonical human proteome that can bind the HLA-A*02:01 allele are identified. The current focus on HLA-A*02:01 allele is due to its high allelic frequency [27], the high accuracy of binding prediction method for complexes involving HLA-A*02:01 [28–31], and availability of crystal structures of complexes between peptide-HLAs and T-cell receptors/TCR-like antibodies. Based on the gene expression data from the TCGA (The Cancer Genome Atlas) cancer samples and adjacent normal tissue samples, and the normal tissue samples from GTEx (Gene Tissue Expression database) [32], cancer-specific peptide-HLA complexes are identified. For each target complex, similar complexes are identified using a combination of sequence similarity and molecular modeling approaches in contrast to just using a sequence similarity based approach as in the case of Expitope webserver [26]. The similar complexes that are expressed in essential, normal tissue samples from the TCGA and GTEx databases are considered potential off-targets. Target complexes are then prioritized based on the number of predicted off-targets associated with them; a target with lower number of off-targets associated with it is assigned higher priority. Due to the computational nature of our work, instead of discarding targets based on cross-reactivity predictions, we use predictions to only prioritize targets for future cell-based validation assays.

In the following section, we describe in detail our strategy for identifying cancer-specific peptide-HLA targets, derived from the canonical human proteome, across various cancer types. This description is followed by results obtained from a comprehensive exploration of the human proteome as well as some specific examples that demonstrate the effectiveness of our approach towards discovering peptide-HLA targets. We conclude with a discussion of the strengths and weaknesses of our strategy in its current form, and possible directions for further improvement.

## Methods

We have developed a computational strategy to identify potential cancer-specific peptide-HLA targets and prioritize these targets based on the likelihood of potential cross-reactivity. Our strategy involves four steps: 1) 9-mers are identified from each protein sequence in the human proteome. 2) Binding affinities of the peptides in complex with the HLA-A*02:01 (most frequent HLA allele [27]) are computed and based on the predicted affinity values, potential peptide-HLA complexes are selected. 3) Cancer-specific peptide-HLA complexes are identified based on the expression of the peptide in cancer versus essential, normal tissue samples. 4) For each of the peptides in the potential cancer-specific complexes, similar peptides in the human proteome are identified and their degree of similarity (DoS) is calculated. The complexes are then prioritized based on the number of similar complexes at different DoS threshold levels. In the subsections below, we describe the individual components of our strategy in more detail.

### Step 1: Extract human proteome

All human proteins and their canonical amino acid sequences are extracted from the reviewed subset of the UniProtKB database [33] (version September 2014). The reviewed subset contains several proteins that correspond to multiple alleles of HLA genes HLA-A, HLA-B, HLA-C, and HLA-DRB1. The proteins that correspond to alleles with highest allelic frequencies are kept as representative of the human proteome and the rest are removed. The genes with highest allelic frequency in European Caucasian US population are HLA-A*02 (29.6 %), HLA-B*07 (14.0 %), HLA-C*07 (16.7 %), and HLA-DRB1*15 (14.4 %) [27]. The reviewed subset also contains entries that correspond to long-intergenic-noncoding RNA (lincRNA) genes [34]; such entries are ignored because lincRNAs are not known to get translated to proteins. In total 20,041 proteins are thus extracted. Since we are interested in identifying peptide-HLA complexes, we extract potential peptides from the set of 20,041 proteins. HLA class I molecules are known to bind peptides that are usually 8 to 10 residue long, although peptides that are longer have also been identified. Among the 2,585 linear epitopes involving HLA-A*02:01 available in the IEDB database [35] (as of February 2015), 1737 (67.2 %) contain peptides that are 9-residue long. In this work, therefore, we only consider peptides that are 9-mers. All overlapping 9-mers are extracted from the protein sequences corresponding to the 20,041 proteins. After removing the 9-mers that contain non-standard amino acids, a set of 11,118,076 peptides remains.

### Step 2: Potential peptide-HLA complexes

Potential peptide-HLA complexes are identified using a webserver called NetMHCstab (version 1.0) [31]. NetMHCstab webserver employs a neural network based machine learning model to predict the binding affinity of a peptide and HLA allele. The predicted binding affinity (output as IC_50_ value in nanomolars) estimates how tightly the peptide and the HLA molecule bind to each other. A peptide is considered a strong binder to a HLA allele if the IC_50_ value is smaller than 50 nanomolars (nM); it is considered a weak binder if the IC_50_ value is smaller than 500 nM. In this work, a peptide and HLA allele is considered a putative complex if the peptide is predicted to bind with an affinity less than 500 nM. The set of 11,118,076 peptides is evaluated for binding affinity with the HLA-A*02:01 allele using the NetMHCstab webserver which results in the identification of 338,452 (3.04 %) potential peptide-HLA complexes.

### Step 3: Cancer-specific complexes

The precise targeting of cancer cells requires that potential peptide-HLA targets are specific to cancer cells [21]. Thus, to evaluate whether or not a peptide-HLA complex can be used for the precise targeting of cancer cells, gene expression data from cancer tissue mRNA samples and essential, normal tissue mRNA samples is compared. In this work any normal tissue that can be sacrificed in the process of treating cancer is classified as non-essential, e.g., tissues from male and female reproductive organs (breast, cervix, fallopian tube, testis, uterus, and vagina). The gene expression data for the cancer tissue samples is derived from TCGA (The Cancer Genome Atlas) repository. The gene expression data for the essential, normal tissues is derived from TCGA as well as GTEx (Genotype-Tissue Expression project) repository [32]. Note that in this work we use normal tissue data from two different sources so that we can ascertain with high confidence the expression levels of a gene in the normal tissues.

Raw data produced from the RNA-Seq analysis [36] of mRNA samples done by TCGA and GTEx is available from the respective data repositories. The data used in this work corresponds to 7,840 TCGA cancer samples (across 27 cancer types), 497 TCGA adjacent normal samples (across 15 essential tissue types), and 2,928 GTEx normal samples (across 22 essential tissue types). The distribution of the samples is tabulated in Additional files 1 and 2. Although the raw data has been processed by TCGA and GTEx to produce gene expression values, we decided to use a common data processing pipeline to exclude any potential discrepancy due to different processing pipelines used by TCGA and GTEx. The data processing pipeline, implemented by Omicsoft [37], has processed the raw RNA-Seq data to produce the gene expression values in each sample, which are measured in Reads Per Kilobase Per Million (RPKM) [38].Using these expression values, the 75-percentile RPKM value of each gene in each cancer type as well as the 75-percentile RPKM value of each gene in each essential, normal tissue type is derived. It has been estimated that depending on the total amount of RNA in the cell, a single copy of a transcript is equivalent to somewhere between 0.5 and 5.0 RPKM [39]. A peptide-HLA complex is, therefore, classified as specific to a cancer type if the 75-percentile RPKM value of the gene (from which peptide is derived) in cancer samples is greater than 5.0, and the maximum of the 75-percentile RPKM across all normal tissue types is less than 0.5. The usage of the above RPKM thresholds implies that, given the 29.6 % allelic frequency of HLA-A*02:01 [27], in 5 % or more cancer patients of a specific type, at least one copy of the target is present in the cancer cells and zero copies in the essential, normal cells. Thus, based on the gene expression values, we restrict discovery of targets to those genes that are highly expressed in cancer cells and weakly expressed in essential, normal tissue cells. Out of the 338,452 potential peptide-HLA complexes, a list of 627 potential cancer-specific complexes is thus obtained.

### Step 4: Off-target complexes

Although we have carefully identified cancer-specific complexes so far, a therapeutic agent targeted at a peptide-HLA complex can have potential off-target effects if similar peptide-HLA complexes are present on the surface of cells from the essential, normal tissues [22, 23]. We have described in the Introduction Section a case study where the off-target toxicity was caused due to the presence of a similar complex in which the amino acids of the peptides were identical at only 5 positions (equivalent to 4 mismatches) [23]. To our knowledge, toxic cross-reactivity due to peptides with more mismatches has not been reported yet. Therefore, we assume in this work, that two peptide-HLA complexes can be considered similar if the HLA alleles are the same and the peptides have 5 or more identical residues at the same positions.

For each of the 627 peptide-HLA complexes, a list of similar peptides in the human proteome (a set of 11,118,076 9-mer peptides) along with the degree of similarity (DoS) between each pair of similar peptides is derived. The DoS between two peptides is defined as the number of identical amino acids in peptides at identical positions, which is same as the hamming distance between the sequence strings of the two peptides [40]. Since the focus of this work is on 9-mer peptides, a maximum DoS value of 9 implies that two peptides are identical. The lower the DoS, the lower the degree of similarity and two peptides are considered dissimilar if DoS is less than 5.

Not all of the similar complexes are however a cause for concern with regard to potential toxicity. If the 95-percentile expression of the similar peptide is greater than 0.5 in any essential, normal tissue type, then we consider that the similar complex will likely result in toxic off-target effects. We have utilized very conservative criteria for determining toxicity so that the risk of not being able to predict potential off-target toxicity is minimal. The target peptide-HLA complexes are prioritized on the basis of the number of associated similar complexes that can potentially result in toxic off-target effects. The higher the number of such similar complexes, the more likely is a toxic off-target effect.

One potential limitation of our approach is that the number of similar complexes is overestimated, because the amino acid positions in the peptides are not discriminated with respect to their importance for binding a particular therapeutic TCR or TCR-like antibody. Amino acid positions in a peptide that are involved in hydrophobic contacts and/or hydrogen bonds with the TCR/antibody are considered important for binding the therapeutic molecule, and the rest are considered non-important [41, 42]. Since this work focuses on peptide-HLA complexes involving HLA-A*02:01, we took advantage of the structures of complexes of peptide-HLA and TCR/antibody in the Protein Data Bank (PDB) [43] to determine the importance of each amino acid position in the peptide, and then incorporated this into our strategy.

There are 32 available crystal structures that involve a 9-mer peptide (containing standard amino acids) with HLA-A*02:01 in complex with a TCR/antibody (see Additional file 3). For any other HLA allele, very few such structures are available. In each of the 32 crystal structures, the important and non-important peptide positions are identified, thus producing a contact pattern corresponding to the 9 positions in the peptide. The hydrophobic contacts and hydrogen bond interactions are identified using software called Chimera [44]. A peptide position is considered to be in hydrophobic contact when the van der Waals distance between any side chain atoms at that position with any atom in the TCR/antibody is between 0.0Å and 0.4Å. Similarly a hydrogen bond is considered to exist between a hydrogen atom connected to an electronegative atom (donor) and another electronegative atom (acceptor) when the distance between the donor and the acceptor is less than 4Å and the angle donor-hydrogen-acceptor is less than 30°. Out of the 32 contact patterns thus identified, only 21 are unique (see Additional file 4). Using these 21 patterns, a simple predictor can classify a position of a query peptide as non-important if fewer than a threshold number of patterns show the position as important. Clearly, the simple predictor is agnostic of the query peptide sequence.

We have developed an improved predictor based on molecular modeling. Given a query peptide, structures of peptide-HLA-A*02:01 complexes are modeled by mutating the amino acids of peptides in the 32 PDB structures to match the query peptide’s amino acid sequence. After mutations are applied, structures are energy minimized to eliminate steric clashes and the structure(s) with any remaining clashes are discarded. Both mutations and energy minimization are done using Chimera in its default settings [44]. Contact patterns are then computed for modeled structures of peptide-HLA-TCR/antibody complexes involving the query peptide and unique patterns are identified. Similar to the simple predictor, a peptide position is classified as non-important if fewer than a threshold number of patterns from the modeled structures show the position as important.

**Fig. 1:**
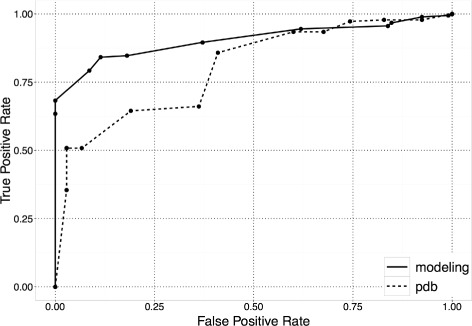
Receiver-operator characteristics of predictors that identify peptide positions which are not important for binding interactions with a therapeutic molecule. A simple PDB-based predictor and a molecular modeling based predictor were compared

We performed a leave-one-out validation experiment [45] to ascertain the Receiver Operator Characteristics (ROC) of the simple predictor and the modeling-based predictor. Each predictor was used to identify non-important peptide positions for known peptide-HLA-TCR/antibody complex based on the remaining known complexes. At different threshold values, the number of true positives (position correctly predicted as non-important), true negatives (position correctly predicted as important), false positives (position incorrectly predicted as non-important), and false negatives (position incorrectly predicted as non-important) were calculated, upon which the true positive rate and false positive rate were calculated. The ROC curves obtained for the simple and modeling-based predictors are shown in Fig. 1. The Area Under Curve (AUC) for the simple predictor is 0.80 and for the modeling-based predictor is 0.90. More importantly, at threshold value equal to 2, the true positive rate is 0.68 and the false positive rate is 0 for the modeling-based predictor.

Clearly, we cannot identify all non-important positions in a peptide, but the ones we identify are the correct ones, at least based on the limited structure data we have so far in PDB. We may still overestimate the importance of certain peptide positions but it allows us to err on the side of caution when predicting similar complexes that can result in potentially toxic off-target effects. In general at least 3 peptide positions are considered to play a role in TCR binding [46], therefore similar complexes with similarity at fewer than 3 important positions are discarded. The peptide-HLA complexes are thus re-prioritized on the basis of the number of remaining similar complexes.

## Results

### potential targets

From UniProtKB database [33], we extracted 20,108 canonical protein sequences that represent the human proteome. The 11,118,076 9-mer peptides derived from the protein sequences were input into the NetMHCstab webserver to compute the binding affinity of each peptide with HLA-A*02:01 [31]. The distribution of the IC50 values of the peptides is shown in Additional file 5. As illustrated in the top panel of the figure, 338,452 peptides were predicted to bind HLA-A*02:01 with IC_50_ less than 500 nM. The distribution of the IC_50_ values of the 338,452 peptides is shown in more detail in the bottom panel of the figure. As illustrated, 104,764 peptides were predicted to bind HLA-A*02:01 with IC_50_ less than 50 nM which is a criterion used to classify a peptide as a strong binder. For the purposes of identifying potential peptide-HLA targets, we further analyzed the cancer specificity of 338,452 and identified 627 potential cancer-specific peptide-HLA targets across 18 TCGA cancer types.

The distribution of the number of targets specific to each cancer type is listed in Table 1. The highest number (72) of targets was identified for skin carcinoma (SKCM) cancer type that is a focus area for the development of therapeutic engineered T-cells [17, 47, 48]. None of the identified targets are specific for tumor types ACC, COAD, KIRC, KIRP, LUAD, PAAD, READ, SARC, and THCA (see Additional file 1 for TCGA tumor type codes). There are 359 unique targets as some of the targets are associated with multiple cancer types. The peptides that constitute the 627 potential targets were derived from 24 unique genes. The distribution of the number of peptides (and unique peptides) derived from each gene is listed in Table 2. Many MAGE (melanoma antigen) family genes are the focus of therapeutic development programs [17, 22, 49] as they are primarily expressed in non-essential testis tissue. Among the 24 genes from which our targets were derived, 8 MAGE genes are present. Since we explored the full human proteome to identify potential peptide-HLA targets, 24 genes have varied subcellular localizations (intracellular, membrane, secreted, etc.). The distribution of the number of targets with respect to the cancer types and genes from which the peptides were derived is listed in Table 3. Genes such as MAGEA3 and MAGEA6 contributed targets for 7 cancer types each, while genes such as UMODL1, NLRP4, MAGEC2, ANKRD30A, and GPRC6A contributed targets for only 1 cancer type.

**Table 1:** Distribution of peptide-HLA targets with respect to different TCGA tumor types

	Tumor	Count
1	SKCM	72
2	LAML	70
3	LUSC	68
4	DLBC	64
5	BLCA	60
6	LIHC	38
7	KICH	35
8	ESCA	31
9	HNSC	31
10	UCS	27
11	PRAD	24
12	BRCA	23
13	CESC	22
14	STAD	22
15	GBM	15
16	LGG	15
17	OV	8
18	UCEC	2

See Additional file 1 for the description of TCGA tumor types

**Table 2:** Distribution of peptide-HLA targets with respect to the genes from which the peptides are derived. The number of unique peptide-HLA targets derived from each gene is also listed

	Gene	Count	Unq.count
1	MAGEA3	84	12
2	MAGEA6	70	10
3	UMODL1	54	54
4	MAGEA9B	52	13
5	NLRP4	47	47
6	MAGEA4	45	9
7	CNTNAP5	35	35
8	MAGEA12	32	16
9	RPE65	30	15
10	ANKRD30A	23	23
11	SMC1B	22	22
12	MAGEA1	22	11
13	MAGEC2	18	18
14	DNTT	16	16
15	TGM4	16	16
16	MAGEA11	13	13
17	TRIM51	11	11
18	DSCR8	10	5
19	EPYC	8	8
20	POTEM	7	7
21	COX7B2	5	5
22	C7orf72	4	4
23	MTRNR2L1	2	2
24	KLKP1	1	1

Some genes provided targets that are specific to multiple cancer types, e.g., MAGEA3 provided 12 unique targets that are specific to 7 different cancer types, equivalent to 84 targets, in total, across 7 cancer types

**Table 3:** Distribution of targets with respect to the cancer type and genes from which the peptides are derived

	Tumor	Gene	Count
1	BLCA	MAGEA12	16
2	BLCA	MAGEA9B	13
3	BLCA	MAGEA3	12
4	BLCA	MAGEA6	10
5	BLCA	MAGEA4	9
6	BRCA	ANKRD30A	23
7	CESC	SMC1B	22
8	DLBC	NLRP4	47
9	DLBC	MAGEA9B	13
10	DLBC	C7orf72	4
11	ESCA	MAGEA3	12
12	ESCA	MAGEA6	10
13	ESCA	MAGEA4	9
14	GBM	RPE65	15
15	HNSC	MAGEA3	12
16	HNSC	MAGEA6	10
17	HNSC	MAGEA4	9
18	KICH	CNTNAP5	35
19	LAML	UMODL1	54
20	LAML	DNTT	16
21	LGG	RPE65	15
22	LIHC	MAGEA3	12
23	LIHC	MAGEA1	11
24	LIHC	MAGEA6	10
25	LIHC	COX7B2	5
26	LUSC	MAGEA9B	13
27	LUSC	MAGEA11	13
28	LUSC	MAGEA3	12
29	LUSC	MAGEA1	11
30	LUSC	MAGEA6	10
31	LUSC	MAGEA4	9
32	OV	EPYC	8
33	PRAD	TGM4	16
34	PRAD	POTEM	7
35	PRAD	KLKP1	1
36	SKCM	MAGEC2	18
37	SKCM	MAGEA12	16
38	SKCM	MAGEA3	12
39	SKCM	TRIM51	11
40	SKCM	MAGEA6	10
41	SKCM	DSCR8	5
42	STAD	MAGEA3	12
43	STAD	MAGEA6	10
44	UCEC	MTRNR2L1	2
45	UCS	MAGEA9B	13
46	UCS	MAGEA4	9
47	UCS	DSCR8	5

See Additional file 1 for the description of TCGA tumor types

### Prioritized targets

**Fig. 2:**
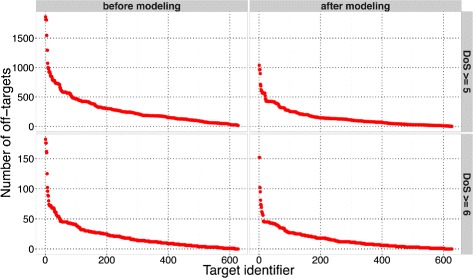
Number of predicted off-targets for each potential peptide-HLA target. The four panels show the number of off-targets predicted based on different DoS threshold levels and based on the usage of the molecular modeling based predictor to identify non-important and important peptide positions. In each panel, the targets are sorted by decreasing y-axis value

The 627 potential targets were prioritized based on the likelihood of off-target toxicity. The targets were first prioritized by the increasing number of associated off-targets at DoS greater than or equal to 6. Further prioritization was similarly done based on similar complexes at DoS greater than or equal to 5. If multiple targets had same number of similar complexes, a target with higher expression in tumor was prioritized higher. As described in the Methods section, we used a molecular modeling based predictor to discriminate important and non-important peptide positions. The prioritized 627 targets are listed in Additional file 6. The number of predicted off-targets associated with each of the 627 targets are shown in Fig. 2, each panel in the figure shows the number of off-targets based on different combinations of usage of molecular modeling based predictor and DoS threshold levels. The figures show that after using the modeling-based predictor, there are fewer off-targets associated with the 627 targets at both DoS ≥ 5 and DoS ≥ 6 threshold levels. Fewer than 100 off-targets are associated with 317 and 624 targets at DoS ≥ 5 and DoS ≥ 6 respectively (right panels in Fig. 2). Out of the 317 and 624 targets, 198 and 614 targets respectively have fewer than 50 off-targets associated with them. Thus, even though there are a few targets with a large number (> 1000) of off-targets associated with them, there are a significant number of targets with reasonable number of associated off-targets that could be experimentally evaluated for cross-reactivity in a tractable manner. It is worth noting that at DoS ≥ 5, there is only 1 target without any associated potential off-target, thus demonstrating the challenge involved in minimizing potential toxic cross-reactivity that could be associated with peptide-HLA targets.

The top-20 peptides that in complex with HLA-A*02:01 allele form the top-20 cancer-specific peptide-HLA targets are listed in Table 4. Each target is listed along with the tumor type for which the target was identified, the gene from which the target peptide was derived, the predicted binding affinity, the 75-percentile expression of the peptide (gene) in the tumor samples corresponding to the tumor type, and the number of predicted off-targets at different DoS threshold levels that can potentially cause toxicity. All of the top-20 targets do not have any associated off-targets at DoS ≥ 6, and have 18 or fewer off-targets at DoS ≥ 5. The 20 targets were identified for 7 tumor types: DLBC, GBM, LAML, LGG, LIHC, LUSC, and SKCM. Peptides from MAGE family genes feature in the top-20 targets list, but the genes are MAGEC2 and MAGEA11 instead of MAGEA3 that has been the focus of most of the research and clinical work on peptide-HLA targets. Perhaps the eluding success with MAGEA3-derived targets is due to the potential cross-reactivity associated with these targets. Although the 12 peptides derived from MAGEA3, which are in the list of 627 targets, include well-investigated peptides such as FLWGPRALV and KVAELVHFL [50], the highest ranked (36^*th*^) peptide is GNWQYFFPV.

**Table 4:** Top 20 peptide-HLA targets prioritized by the number of associated off-targets

	Tumor	Peptide	Gene	IC_50_	RPKM.tumor	Sim.5	Sim.6
1	DLBC	HLSPIDCEV	NLRP4	42.48	6.57	0	0
2	LAML	LTSMWSPAV	UMODL1	279.13	6.47	1	0
3	DLBC	HLDHPHPAV	NLRP4	147.43	6.57	2	0
4	SKCM	SLSVMSSNV	MAGEC2	265.87	19.91	3	0
5	DLBC	MMAWSDNKI	NLRP4	90.11	6.57	3	0
6	LIHC	TQIGIEWNL	COX7B2	229.74	19.13	4	0
7	DLBC	CLFEMQDPA	NLRP4	37.51	6.57	5	0
8	LAML	YLSHPSCNV	UMODL1	16.04	6.47	5	0
9	LIHC	GIEWNLSPV	COX7B2	361.90	19.13	7	0
10	LUSC	GLGCSPASI	MAGEA11	471.75	10.52	7	0
11	LGG	RQAFEFPQI	RPE65	456.69	6.60	7	0
12	GBM	RQAFEFPQI	RPE65	456.69	6.30	7	0
13	DLBC	GMWTDTFEF	NLRP4	172.47	6.57	10	0
14	SKCM	YLNWQDTAV	TRIM51	10.30	7.35	11	0
15	LUSC	VLWGPITQI	MAGEA11	24.07	10.52	15	0
16	DLBC	TLDHTGVVV	NLRP4	497.98	6.57	15	0
17	LGG	TMGVWLHIA	RPE65	314.41	6.60	16	0
18	GBM	TMGVWLHIA	RPE65	314.41	6.30	16	0
19	SKCM	KVWVQGHYL	MAGEC2	407.64	19.91	18	0
20	LAML	KINCNNFRL	UMODL1	304.37	6.47	18	0

The table lists the peptides which in complex with HLA-A*02:01 form the targets, the cancer types which specifically express the targets, the genes from which the peptide were derived, the predicted binding affinities (IC_50_ in nanomolars) of the target complexes, the 75% expression (in RPKM) of genes in the tumor samples, and the number of potential off-targets at DoS ≥ 5 (Sim.5) and DoS ≥ 6 (Sim.6). See Additional file 1 for the description of TCGA tumor types

### Top targets for 3 different cancer types

**Fig. 3:**
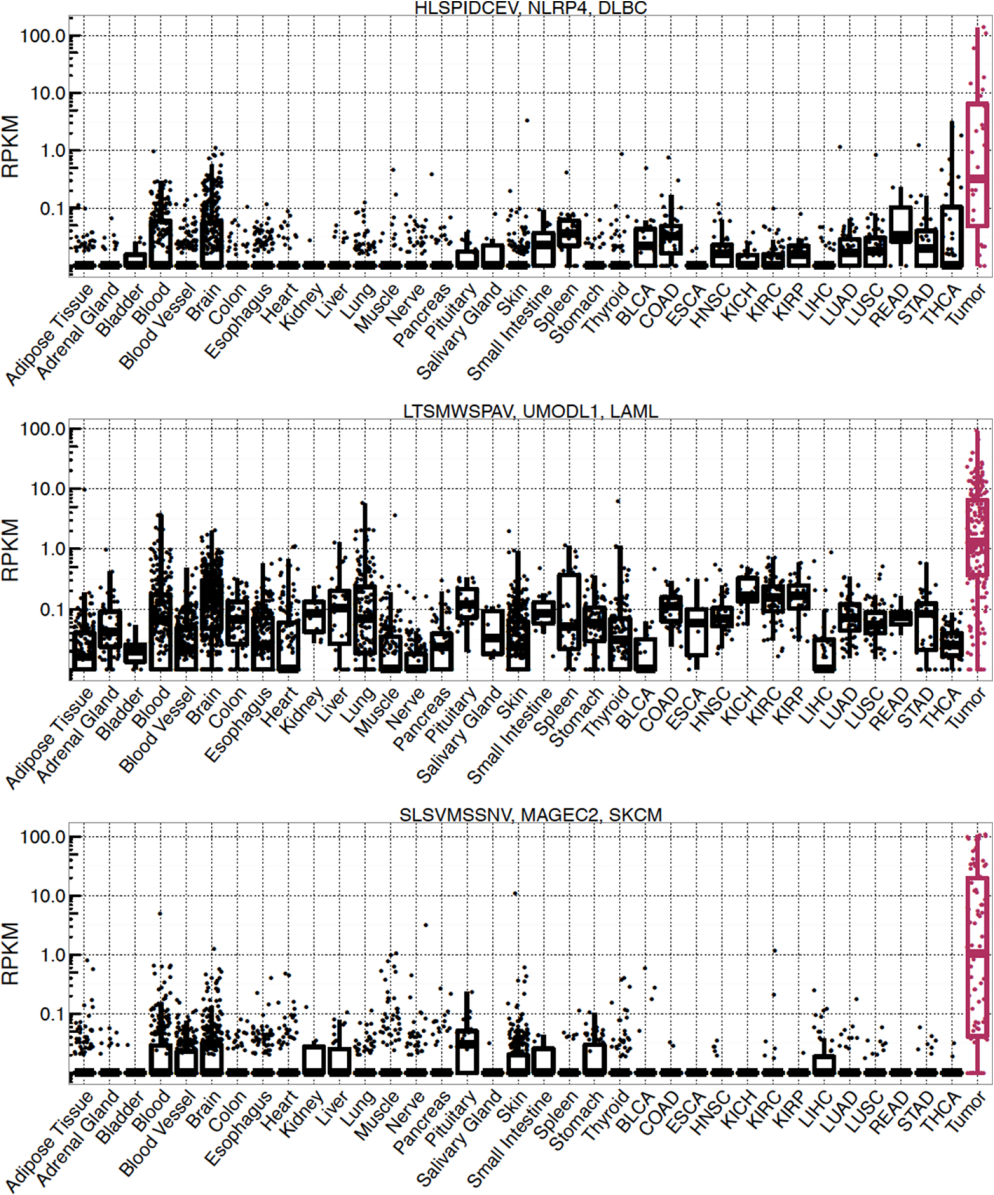
Cancer and essential, normal tissue expression of the top target peptides for three different cancer types. Each dot represents the RPKM value of the peptide/gene in one sample. The essential, normal tissue samples are shown in black color and the cancer samples are shown in magenta color. See Additional file 1 for the description of TCGA tumor types

The top targets were identified for Diffuse Large B-Cell Lymphoma (DLBC), Acute Myeloid Leukemia (LAML), and Skin cutaneous melanoma (SKCM) cancer. These targets are peptides HLSPIDCEV (ranked 1 ^*st*^), LTSMWSPAV (ranked 2 ^*nd*^), and SLSVMSSNV (ranked 4^*th*^) respectively. Following are the three genes from which the peptides were derived: NLRP4 (NACHT, LRR and PYD domains-containing protein 4), UMODL1 (Uromodulin-like 1), and MAGEC2 (Melanoma-associated antigen C2). Expression of the peptides (genes) in each GTEx normal sample, TCGA normal sample, and tumor samples (from the tumor type associated with the targets) is shown in Fig. 3. As illustrated, expression of the peptides in normal samples is very low compared to the expression in the tumor samples. Through structural modeling based analysis of contact patterns, positions {1,5,7}, {1,4,5}, and {4,5,8} were classified as important for the peptides HLSPIDCEV, LTSMWSPAV, and SLSVMSSVNV respectively.

The predicted off-targets associated with two of the three targets (HLSPIDCEV has no predicted associated off-target) are listed in Table 5. The table lists peptides that are similar to the target peptide, binding affinities of the similar peptides to HLA-A*02:01, genes from which the similar peptides were derived, and top 2 normal tissue samples each from GTEx and TCGA that express these genes at a level which could result in toxic cross-reactivity. The peptide LTSMWSPAV (DLBC target) has 1 associated off-target that was derived from the gene DNAH7 (Dynein heavy chain 7, axonemal). The expression levels of DNAH7 in essential, normal tissue samples in GTEx and TCGA databases suggest potential concern for toxic cross-reactivity in brain, lung, and other tissues. The peptide SLSVMSSNV (SKCM target) has 3 associated off-targets that were derived from the genes SLC31A2 (Solute carrier family 31, member 2), OTOP2 (Otopetrin 2), and OTOP3 (Otopetrin 2). The expression levels of these genes in essential, normal tissue samples suggest potential concern for toxic cross-reactivity in small intestine, colon, and other tissues.

**Table 5:** Predicted off-targets associated with top LAML and SKCM specific targets

	Tumor	Target	Target.gene	Off.target	IC_50_	Gene	Normal.tissue.samples	
1	LAML	LTSMWSPAV	UMODL1	LLKMWFPEV	21.60	DNAH7	Pituitary, Brain, LUAD, LUSC	
2	SKCM	SLSVMSSNV	MAGEC2	MLAVMSYNT	206.18	SLC31A2	Blood, Salivary Gland, HNSC, LUAD	
				MLYVMWKNV	137.41	OTOP2	Colon, S. Intestine, COAD, READ	
				VLFVMWKNV	276.13	OTOP3	Esophagus, S. Intestine, STAD, HNSC	

The table lists the off-target peptides, binding affinity (IC_50_ in nanomolars) of peptide-HLA-A*02:01 complexes involving the off-target peptides, genes from which the off-target peptides were derived, and the essential, normal tissue types from GTEx and TCGA with high expression of the off-target peptides/genes. See Additional file 1 for description of TCGA tumor types

### MAGEA3 peptide KVAELVHFL

Fatal adverse events were reported in the clinical trial involving engineered T-cell based therapy against the MAGEA3 peptide KVAELVHFL-HLA-A*02:01 complex [17]. Subsequently, 9 different peptides very similar to the target peptide were experimentally evaluated as potential off-targets and peptide KMAELVHFL from the gene MAGEA12 was implicated as the most likely off-target. Using our strategy, we identified 6 (including the MAGEA12 peptide) out of the 9 peptides as potential off-targets that are listed in Table 6. Peptide KVVSLVHFL from MAGEB18 was not identified because expression of MAGEB18 in essential, normal tissues is too low for the peptide to be recognized as an off-target by our strategy. The other two peptides that were not identified are GIAELVHFS (from gene PPP2R1B) and TVAELVQFV (from gene MAGEF1). These two peptides are not contained in the canonical human proteome sequences derived from UniProtKB and were therefore not identified. Although we identified KVAELVHFL as a potential SKCM target, it was found to be associated with 273, 30, 12, and 5 potential off-targets at DoS ≥ 5, 6, 7, and 8 respectively. Therefore, it was ranked 516^*th*^ out of the 627 potential targets making it a very low-priority target.

**Table 6:** Predicted off-targets associated with target KVAELVHFL-HLA-A*02:01 derived from MAGEA3

	Off.target	DoS	IC_50_	Off.target.gene	Normal.tissue.samples
1	KVAELVHIL	8	103.72	DDX28	Muscle, Skin, BLCA, HNSC
2	KVAELVQFL	8	46.07	MAGEC3	Brain, Spleen, THCA, ESCA
3	KMAELVHFL	8	2.66	MAGEA12	Brain, Muscle, BLCA, HNSC
4	SAAELVHFL	7	187.05	EPS8L2	Esophagus, Kidney, HNSC, STAD
5	KLEELVHFL	7	11.98	MRVI1	Blood Vessel, Blood, ESCA, STAD
6	SAADLVHFL	6	211.83	EPS8	Blood Vessel, Adipose Tissue, COAD, READ

The table lists the off-target peptides, degree of similarity of the off-target peptides with the target peptide, binding affinity (IC_50_ in nanomolars) of peptide-HLA-A*02:01 complexes involving the off-target peptides, genes from which the off-target peptides were derived, and the essential, normal tissue types from GTEx and TCGA with high expression of the off-target peptides/genes

### CTAG1A/NY-ESO-1 peptide SLLMWITQC

The cancer testis antigen 1 A (CTAG1A, also known as CTAG1B/NY-ESO-1) peptide SLLMWITQC in complex with HLA-A*02:01 is an active target for engineered T-cell based therapy in multiple myeloma, synovial sarcoma, and advanced melanoma [51]. In phase I/II clinical trials, the safety of the therapy targeting the CTAG1A peptide has been demonstrated [52]. This target was not in our list of 627 targets because the 75-percentile expression of CTAG1A/CTAG1B in the cancer samples from each TCGA cancer type is less than 5 RPKM (we recognized a peptide as a target for a particular cancer type if the 75-percentile expression of the peptide/gene was greater than 5 RPKM). As described earlier, our aim with using the 75-percentile expression values was to identify targets that could be present in at least 5 % of the TCGA cancer patients of a specific type. But even if our comprehensive analysis of the human proteome might miss targets that can exist in a smaller subset of cancer patients, our strategy could still be used to predict the likelihood of cross-reactivity associated with any peptide-HLA target. Fortunately, multiple structures of this particular target in complex with TCR are available in the Protein Data Bank. These structures inform that peptide positions 4, 5, 6, 7, and 8 are important for TCR binding. Incorporating this information into our strategy, we identified 18 off-targets at DoS ≥ 5 and 1 off-target at DoS ≥ 6 that are listed in Table 7. If peptide SLLMWITQC were a part of our target list, it would have been ranked 51 ^*st*^ from the top. The high ranking of the target due to fewer predicted off-targets thus demonstrates the ability of our strategy to correctly prioritize a target that has not been associated with any toxic off-target effects in clinical trials to date.

**Table 7:** Predicted off-targets associated with target SLLMWITQC-HLA-A*02:01 derived from CTAG1A/NY-ESO-1

	Off.target	DoS	IC_50_	Off.target.gene	Normal.tissue.samples
1	WLLPWICQC	6	66.56	GRID1	Brain, Thyroid, THCA, STAD
2	SLVKPITQL	5	206.18	ITGAM	Blood, Spleen, LUAD, LUSC
3	QLLMGIEQA	5	492.62	CABIN1	Blood, Blood Vessel, THCA, STAD
4	FLLHWITRG	5	335.50	NPC1L1	Liver, Small Intestine, STAD, LIHC
5	SILMYITSL	5	67.65	DMD	Muscle, Nerve, BLCA, STAD
6	PLLYNITQV	5	321.29	PHTF2	Muscle, Blood, HNSC, BLCA
7	TLLMVITGV	5	13.86	KIRREL2	Pancreas, Stomach, KIRP, KICH
8	LLTMHITQL	5	133.75	FBXL22	Colon, Blood Vessel, BLCA, STAD
9	FLLMFIKQL	5	37.51	LRBA	Pituitary, Skin, KIRC, KICH
10	MLLMKIQQL	5	62.71	SIMC1	Adrenal Gland, Muscle, ESCA, STAD
11	SLVYPITQV	5	30.87	BNC1	Salivary Gland, Esophagus, HNSC, STAD
12	RLLQVITQT	5	236.04	DNAH1	Blood, Liver, ESCA, LUAD
13	GLLNWITGA	5	13.06	VWA5B1	Pituitary, Bladder, KIRP, KIRC
14	SLSMGITLI	5	72.18	SLC16A14	Brain, Spleen, HNSC, LIHC
15	SALDQITQV	5	423.37	DNAH2	Lung, Brain, LUAD, LUSC
16	SILVWIFQA	5	52.18	SYNGR4	Brain, Pancreas, STAD, COAD
17	SLSKKITQV	5	59.73	CCDC38	Liver, Spleen, LIHC, HNSC
18	FLNRWITFC	5	52.74	IL2	Bladder, Small Intestine, STAD, BLCA

The table lists the off-target peptides, degree of similarity of the off-target peptides with the target peptide, binding affinity (IC_50_ in nanomolars) of peptide-HLA-A*02:01 complexes involving the off-target peptides, genes from which the off-target peptides were derived, and the essential, normal tissue types from GTEx and TCGA with high expression of the off-target peptides/genes

## Discussion

In this paper, we have described a novel computational strategy to identify potential cancer-specific peptide-HLA complexes that can be targeted by therapeutics such as engineered T-cells and “TCR-like” antibodies [8, 8–11, 16–18]. The strength of our strategy lies in not only identifying peptide-HLA targets but also in estimating the potential toxic cross-reactivity that could result from therapeutic action against such targets. After a comprehensive analysis of the canonical human proteome, we identified 627 peptide-HLA-A*02:01 targets that are specific to 18 different TCGA cancer types. Only those peptides that are highly expressed in cancer samples, and have extremely low expression in essential, normal tissue samples were considered potential targets. Peptides similar to the target peptide were identified from the human proteome based on the similarity of residues. We introduced a molecular modeling-based predictor that classifies peptide positions as important or non-important for interacting with potential therapeutic molecules, and used the predictor to better estimate peptide similarity. The targets were prioritized based on the number of peptides in the human proteome that are similar to the target peptides and are also expressed in essential, normal tissue samples.

At different levels of peptide similarity, measured as the degree of similarity (DoS) value, each target peptide is associated with a different number of potential off-targets (similar peptides). The higher the DoS value, the fewer is the number of similar peptides. The list of Top-20 prioritized target peptides (see Table 4) shows that although at DoS ≥ 6 threshold level, there is no associated off-target, at DoS ≥ 5 there are more than 1 off-targets except in the case of the topmost target. We earlier discussed an off-target peptide ESDPIVAQY from Titin (TTN) that was implicated in fatal cardiac toxicity [22]. The DoS of the target and the off-target peptide is 5, which informs us that a peptide that is similar at 5 identical amino acid positions cannot be disregarded as a potential off-target. Therefore, our finding that almost all the potential targets could have off-targets at DoS ≥ 5 emphasizes the challenge involved in developing therapeutics targeting peptide-HLA complexes.

Given the challenges involved, our comprehensive analysis of the canonical human proteome for identifying novel cancer-specific peptide-HLA targets prioritized by likelihood of toxic cross-reactivity, could prove to be an important step in the development of therapeutic molecules with low cross-reactivity. We have demonstrated the ability to predict the potential toxic cross-reactivity that was observed in the clinical trial involving administration of engineered T-cells targeting MAGEA3 peptide KVAELVHFL in complex with HLA-A*02:01 [17]. Based on our analysis, this MAGEA3 peptide may not have been investigated in a clinical trial.

The current work is focused on targets that involve peptides in complex with HLA-A*02:01. This gives us two benefits: 1) the binding affinity predictions are most accurate for the complexes involving HLA-A*02:01 [29, 31], and 2) the availability of crystal structures of the complexes involving HLA-A*02:01 allows us to accurately identify the peptide positions that are important or not important for the binding interactions with a T-cell receptor (TCR) or antibody. To predict potential off-targets, we identify peptides in the human proteome that are similar to a target peptide, two peptides are more similar if they have more amino acids identical in identical peptide positions. The identification of non-important positions allows us to ignore a peptide as a candidate off-target if it is mostly similar at positions that are not important for the binding interactions with TCR or antibody. Thus, we avoid overestimating the number of the off-target peptides to some extent.

Although the strategy described here is focused on HLA-A*02:01, it can be used to predict cross-reactivity of complexes involving any other HLA allele if binding affinities of complexes can be estimated. We slightly modified our strategy to make cross-reactivity predictions for the MAGEA3 peptide EVDPIGHLY-HLA-A*01:01 complex [22]. In the Step 2 of our strategy, NetMHCstab webserver was used to predict binding affinities for all 9-mer peptides (from the human proteome) in complex with HLA-A*01:01 instead of HLA-A*02:01 [31]. Since very few crystal structures of peptide-HLA-A*01:01 complexes are available, it was not possible to discriminate important and not-important peptide positions. We identified 68 potential off-targets that are expressed in essential, normal tissues and could result in off-target toxicity. Even though the number 68 could be an overestimate because we were not able to discriminate the importance of peptide positions, most importantly, the peptide ESDPIVAQY from Titin (TTN) was identified as the off-target with the highest expression in essential, normal tissues among the 68 potential off-targets.

There are some limitations to our strategy, several of which could be addressed in future work. 1) It is limited to 9-mer peptides and HLA-A*02:01, but can be easily expanded in the future by taking advantage of the peptide-HLA binding prediction algorithms that can handle peptides of varied lengths and different HLA alleles. 2) It employs computational predictions of peptide-HLA binding affinities, although we consider even a weakly binding peptide (predicted IC_50_ < 500 nM) as a potential complex, we could still miss some potential peptides that are predicted to be non-binders, but actually are binders. 3) It ignores two important components of the mechanism by which a peptide gets presented at the cell surface by a HLA molecule. Proteasomal cleavage and TAP (Transporter associated with antigen processing)-peptide binding are critical for peptide presentation along with peptide-HLA binding [53]. There are a few computational algorithms available that predict proteasomal cleavage likelihood of a peptide [54] and TAP-peptide binding [55, 56], but these algorithms are less accurate. However, development of improved algorithms in the future would allow us to incorporate them in our strategy. 4) It explores the full canonical human proteome to identify targets and off-targets, which means that any peptide that is part of gene isoforms [57] or common allelic variations is ignored. Our future work will address this issue by exploring the full human proteome in more detail, however it needs to be emphasized that even if we analyze the full proteome by including every known isoform, a patient can have novel isoforms of a protein (novel peptides) that have not been discovered so far.

It needs to be emphasized that the targets and the associated off-targets that we have discovered require experimental validation which is beyond the scope of our purely in-silico work. Given the computational limitations described above, a subset of the targets and associated off-targets may not be validated. That is precisely the reason we have not filtered out any potential target based on the cross-reactivity analysis. Instead we have only used it to prioritize potential cancer-specific targets and have purposefully erred on the side of caution.

## Conclusions

To date, a simple two-step strategy has been used to discover cancer-specific peptide-HLA targets for engineered T-cell or antibody therapy. Such a strategy involves the discovery of cancer-specific genes in the first step followed by the identification of the HLA binding peptides in the next step. Virtually no attention is given to the likelihood of toxic cross-reactivity which has led to the focus on MAGEA3 and other cancer-testes antigen genes that are not expressed in essential, normal human tissues. However, clinical trials have shown the dangers involved in not considering the potential off-targets in therapies involving peptide-HLA targets. Our strategy provides a unique approach to discovering cancer-specific peptide-HLA targets for engineered T-cell or antibody therapy. The prioritized target list derived from the comprehensive exploration of the human peptidome presents a fresh starting point for the systematic discovery of specific peptide-HLA targets in various cancer types which will hopefully lead to the development of therapies with minimal off-target toxicity.

## Abbreviations

BLAST, basic local alignment search tool; DoS, degree of similarity; GTEx, gene tissue expression; HLA, human leukocyte antigen; IEDB, immune epitope database; MHC, major histocompatibilty complex; PDB, protein data bank; RPKM, reads per kilobase per million; ROC, receiver operator characteristics; TCGA, The Cancer Genome Atlas; TCR, T-cell receptor

## Additional files

Additional file 1 Distribution of TCGA tumor samples. (TIF 729 kb)

Additional file 2 Distribution of GTEx essential, normal tissue samples and TCGA essential, adjacent normal tissue samples. Breast, Cervix, Fallopian tube, Uterus, Vagina. (TIF 473 kb)

Additional file 3 Protein Data Bank (PDB) IDs for structures of complexes between peptide-HLA-A*02:01 and T-cell receptor/Antigen binding domain of antibody. (TIF 574 kb)

Additional file 4 21 unique contact patterns derived from the 32 structures listed in Table S3 (see Additional File 3). Each of the 9 peptide positions was evaluated for contact with the T-cell receptor/ Antigen binding domain of the antibody in each of the 32 complexes. A cross symbol means that the residue at the peptide position is not in contact. (TIF 1023 kb)

Additional file 5 Predicted binding affinities of peptide-HLA-A*02:01 complexes involving all 9-mers from the canonical human proteome. The top panel shows the distribution of the binding affinities of all complexes, and the bottom panel shows the distribution of the binding affinities of the complexes with predicted IC_50_ < 500 nM. (TIF 256 kb)

Additional file 6 627 peptide-HLA targets prioritized by the number of associated off-targets. (XLSX 79 kb)
