# Supplementary figures and images for: In-silico discovery of cancer-specific peptide-HLA complexes for targeted therapy
Source: BMC Bioinformatics. 2016 Jul 20;17:286. doi: 10.1186/s12859-016-1150-2 (PMC4955262; doi:10.1186/s12859-016-1150-2)

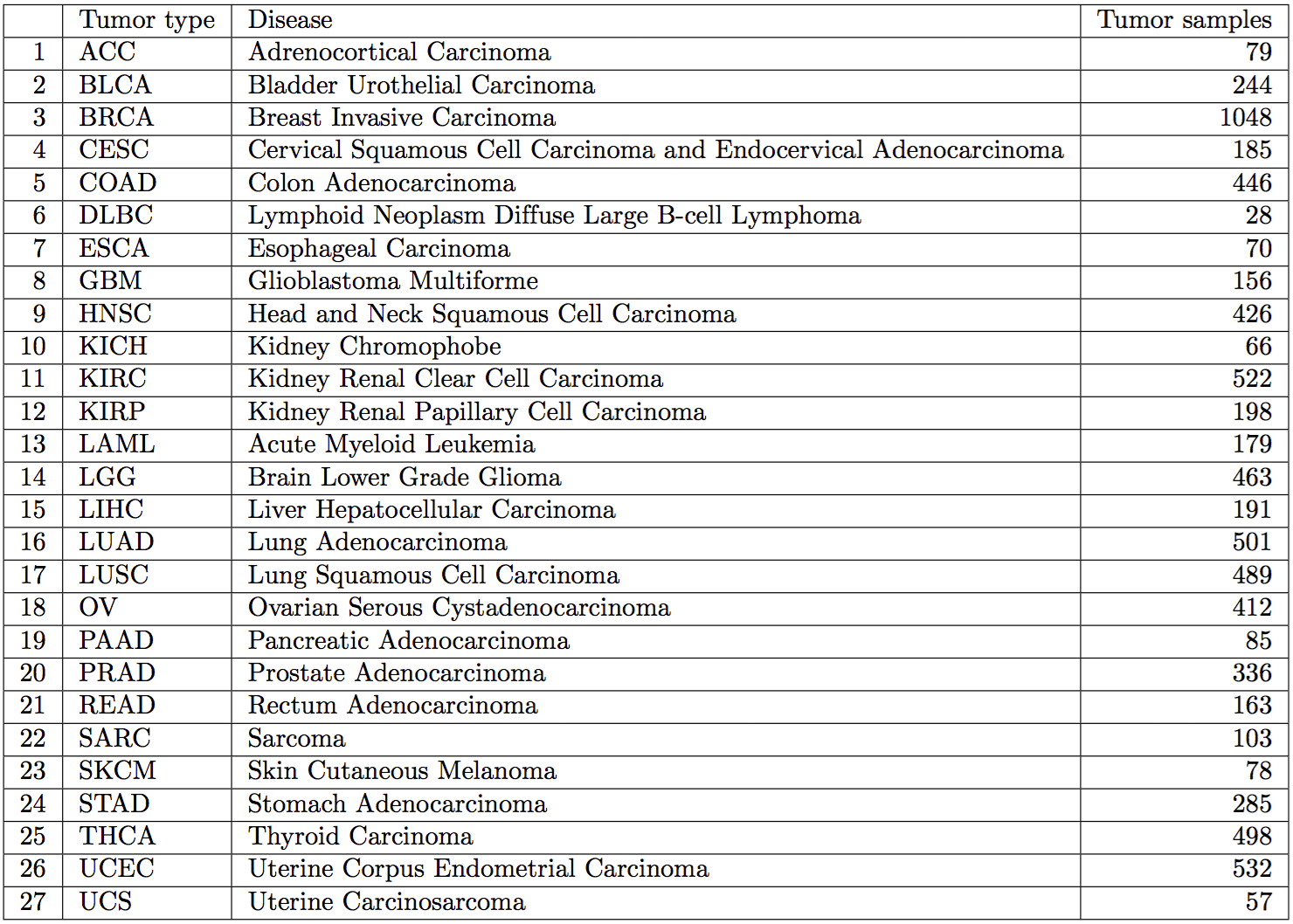

Supplement: Additional file 1 — Distribution of TCGA tumor samples. (TIF 729 kb) [file 12859_2016_1150_MOESM1_ESM.tif]

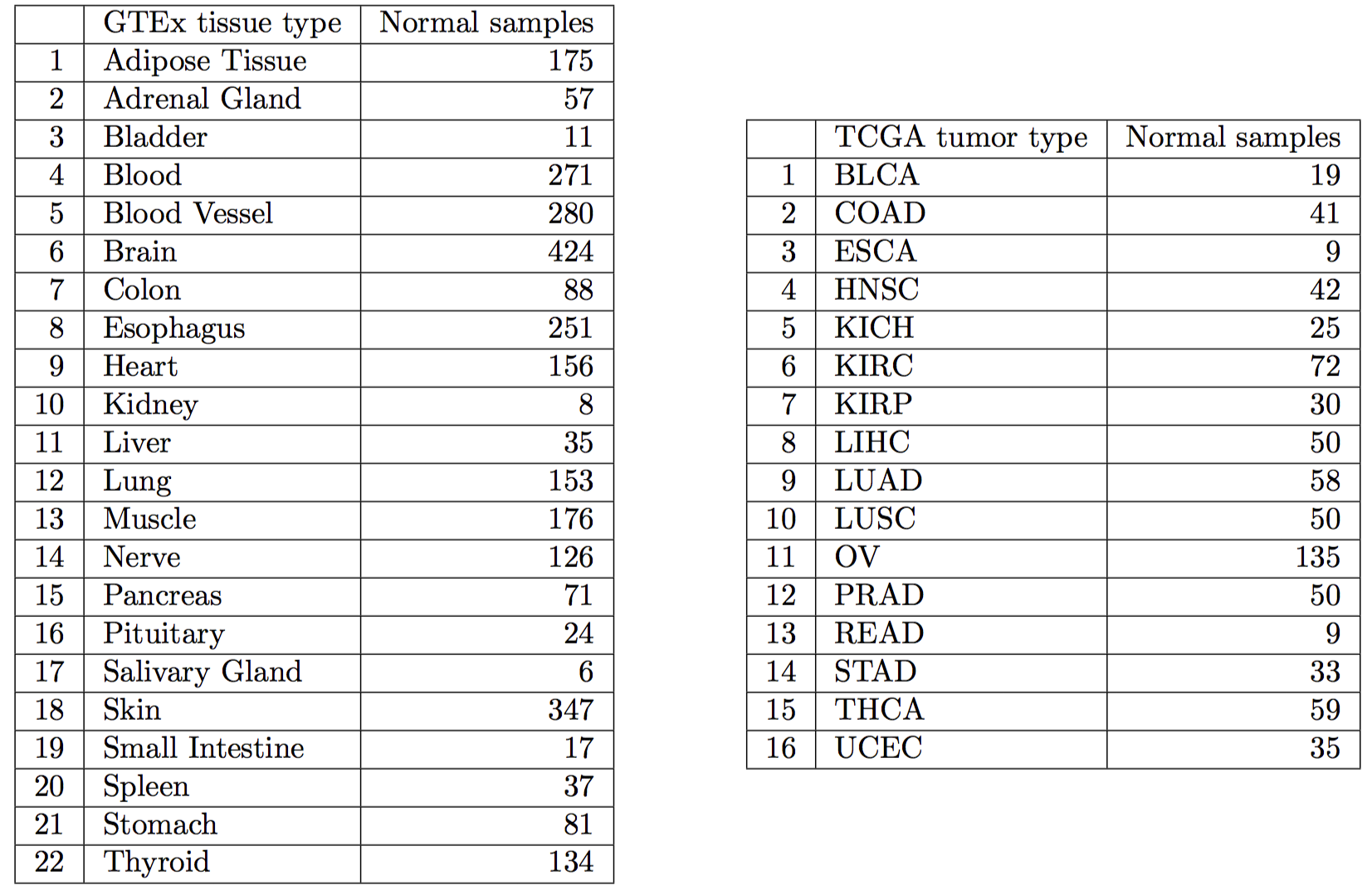

Supplement: Additional file 2 — Distribution of GTEx essential, normal tissue samples and TCGA essential, adjacent normal tissue samples. Breast, Cervix, Fallopian tube, Uterus, Vagina. (TIF 473 kb) [file 12859_2016_1150_MOESM2_ESM.tif]

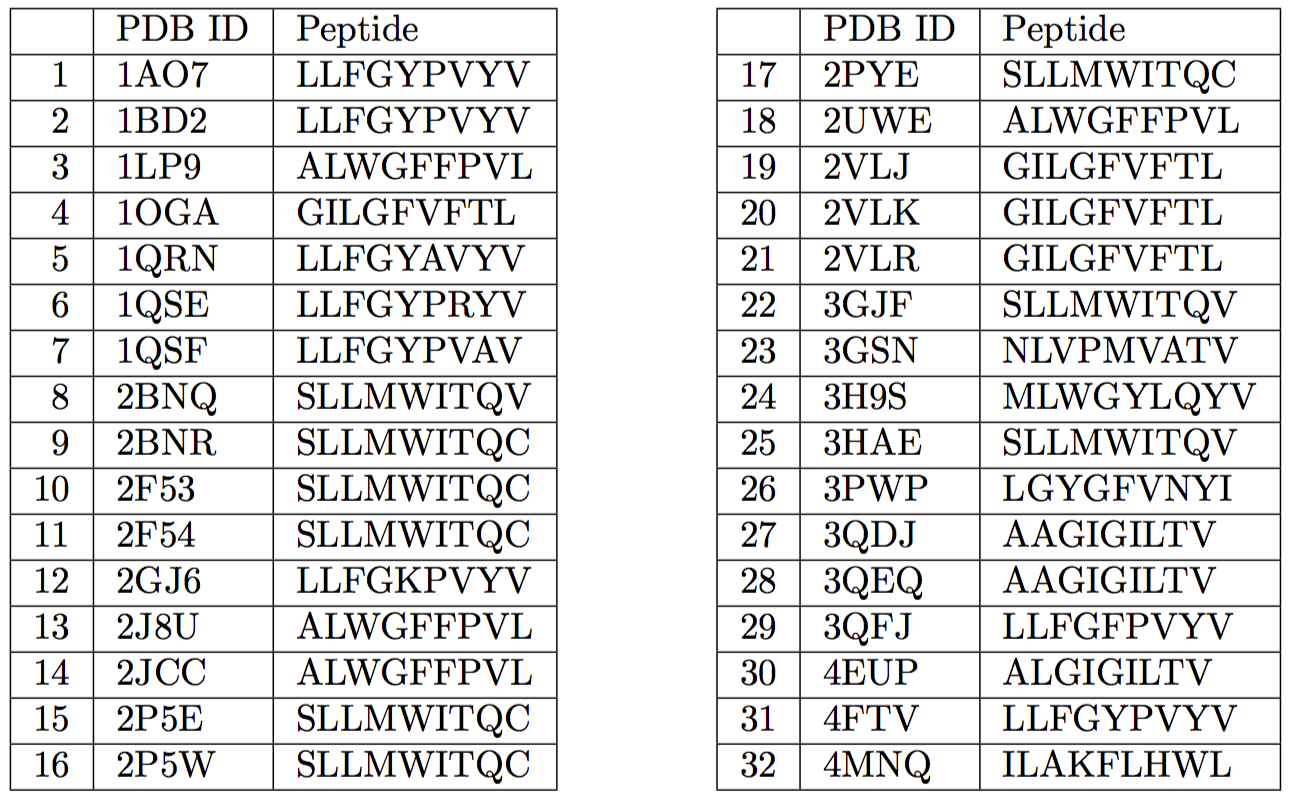

Supplement: Additional file 3 — Protein Data Bank (PDB) IDs for structures of complexes between peptide-HLA-A*02:01 and T-cell receptor/Antigen binding domain of antibody. (TIF 574 kb) [file 12859_2016_1150_MOESM3_ESM.tif]

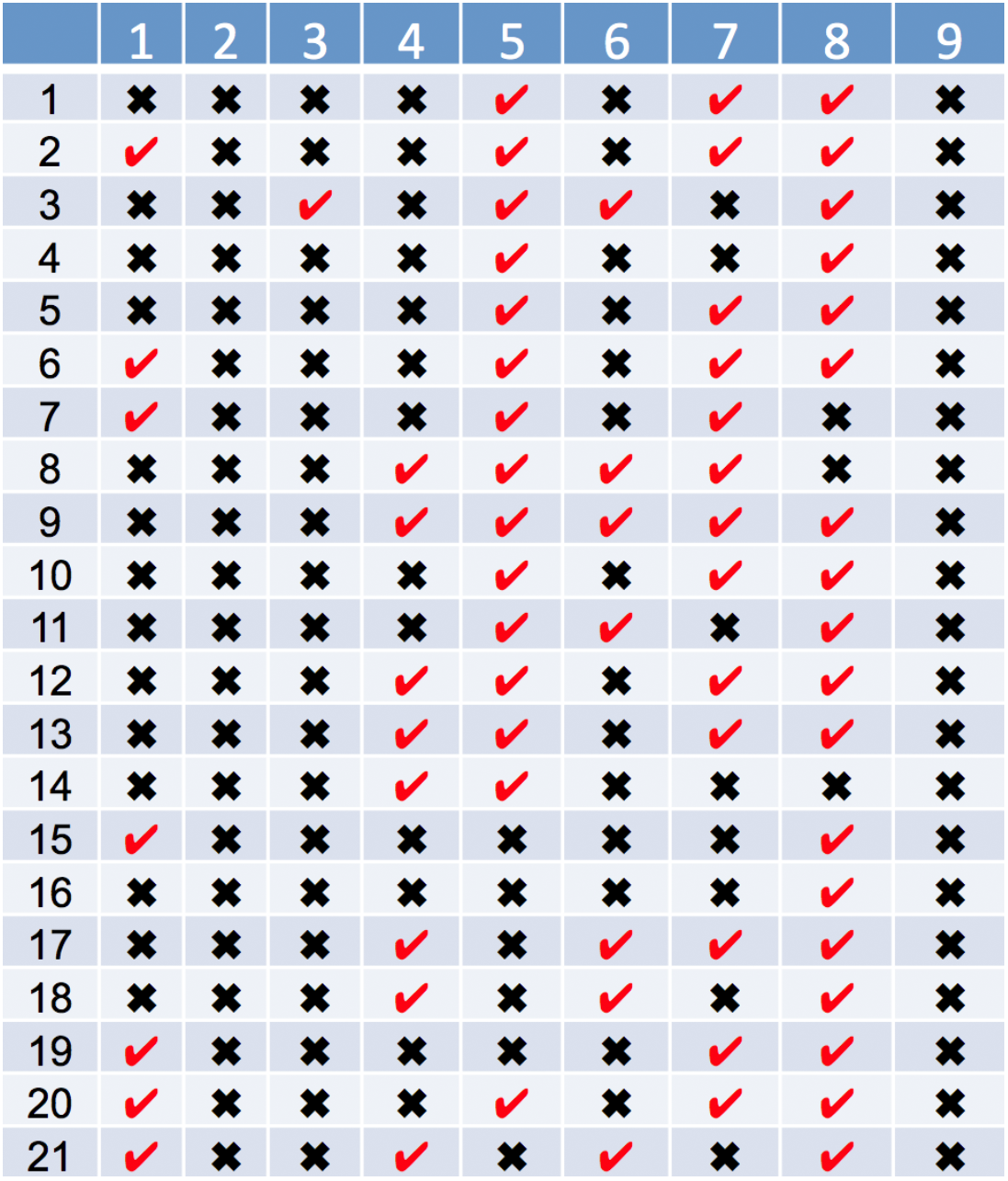

Supplement: Additional file 4 — 21 unique contact patterns derived from the 32 structures listed in Table S3 (see Additional File 3). Each of the 9 peptide positions was evaluated for contact with the T-cell receptor/ Antigen binding domain of the antibody in each of the 32 complexes. A cross symbol means that the residue at the peptide position is not in contact. (TIF 1023 kb) [file 12859_2016_1150_MOESM4_ESM.tif]

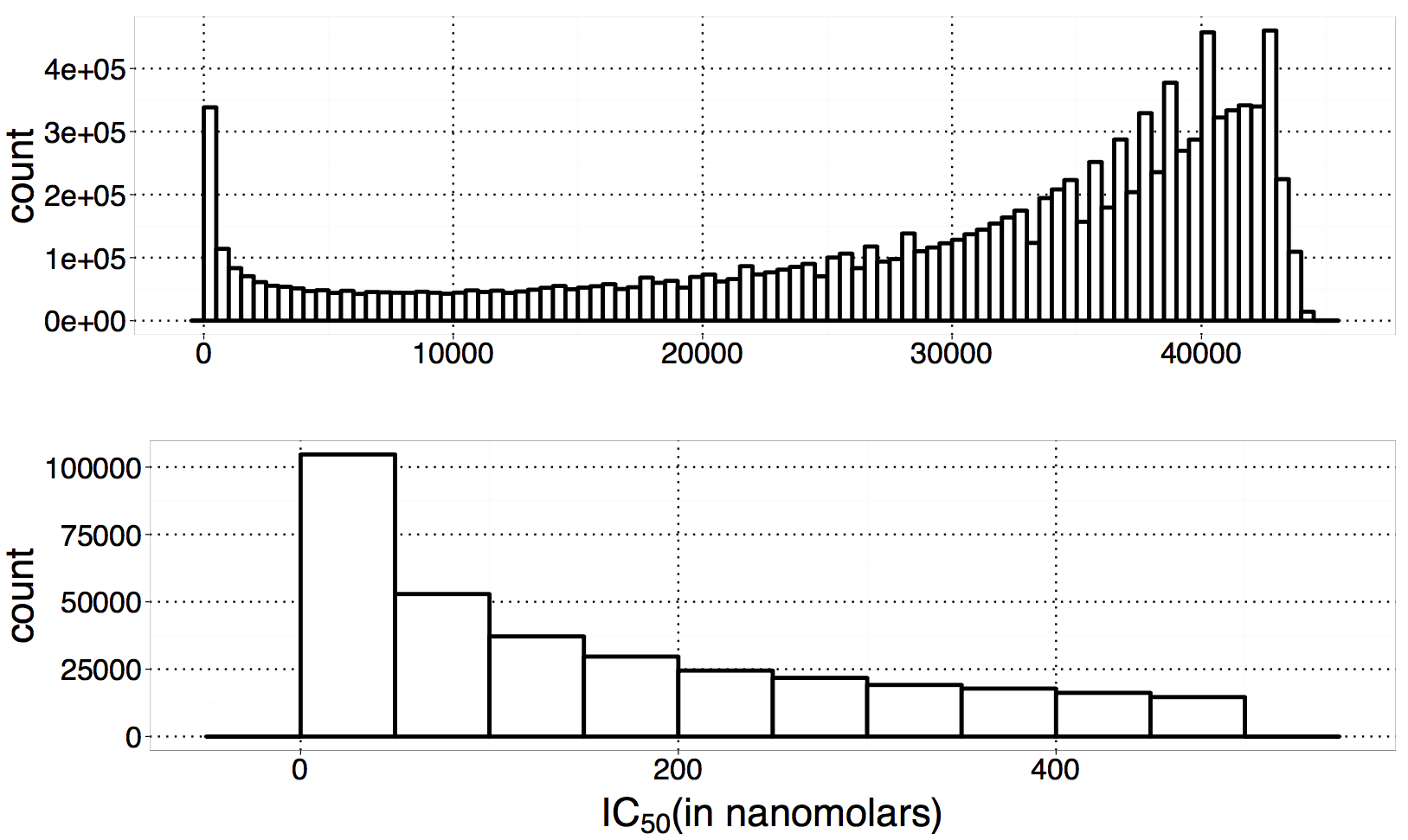

Supplement: Additional file 5 — Predicted binding affinities of peptide-HLA-A*02:01 complexes involving all 9-mers from the canonical human proteome. The top panel shows the distribution of the binding affinities of all complexes, and the bottom panel shows the distribution of the binding affinities of the complexes with predicted IC50 < 500 nM. (TIF 256 kb) [file 12859_2016_1150_MOESM5_ESM.tif]
